# Proline‐Serine–Threonine Phosphatase‐Interacting Protein 2 Alleviates Diabetes Mellitus‐Osteoarthritis in Rats through Attenuating Synovial Inflammation and Cartilage Injury

**DOI:** 10.1111/os.13000

**Published:** 2021-05-03

**Authors:** Ming Li, Yan‐bo Xiao, Xin‐tao Wang, Jin‐peng Zhuang, Chang‐long Zhou

**Affiliations:** ^1^ Department of Orthopaedics The 2nd Affiliated Hospital of Harbin Medical University Harbin China; ^2^ Heilongjiang University Hospital Harbin China

**Keywords:** PSTPIP2, Diabetes mellitus, Osteoarthritis, Synovium, Cartilage

## Abstract

**Objective:**

To explore the possible way of proline‐serine–threonine phosphatase‐interacting protein 2 (PSTPIP2) influencing diabetes mellitus‐osteoarthritis (DM‐OA) progression.

**Methods:**

*In vivo*, eight‐week‐old male Sprague Dawley rats were induced with DM‐OA by intraperitoneal injection of streptozotocin with high‐fat diet feeding and intra‐articular injection of monoiodoacetate. PSTPIP2 overexpression was achieved by intra‐articular injection of lentivirus vectors. PSTPIP2 expression was verified by real‐time polymerase chain reaction and Western blotting. Histological changes were examined by hematoxylin/eosin and safranin‐O/fast‐green staining. *In vitro*, rat synovial fibroblasts were induced DM‐OA by stimulation of high glucose (HG) and interleukin (IL)‐1β. PSTPIP2 overexpression was achieved by lentivirus infection. U0126 was added as an ERK inhibitor. Levels of tumor necrosis factor (TNF)‐α, IL‐6, and IL‐1β were detected using enzyme‐linked immunosorbent assay. Expression of matrix metalloproteinase (MMP)‐3, MMP‐13, aggrecanase‐2 (ADAMTS‐5), intercellular cell adhesion molecule (ICAM)‐1, extracellular regulated protein kinase (ERK) and phospho‐ERK (p‐ERK) was detected by Western blotting.

**Results:**

In DM‐OA rats, PSTPIP2 relative messenger RNA (mRNA) level was significantly decreased compared to control rats. The protein expression was also decreased obviously. Inflammation score in synovium was dramatically increased, accompanying with increased TNF‐α, IL‐6, and IL‐1β levels. Osteoarthritis research society international (OARSI) score in cartilage was markedly increased, along with increased MMP‐3, MMP‐13, ADAMTS‐5, ICAM‐1, ERK and p‐ERK expression. In PSTPIP2‐overexpressed DM‐OA rats, PSTPIP2 mRNA level and protein expression was increased compared to DM‐OA rats received negative‐control lentivirus vectors. The inflammation score, as well as TNF‐α, IL‐6, and IL‐1β levels were dramatically decreased. Also, the OARSI score and protein expression of MMP‐3, MMP‐13, ADAMTS‐5, ICAM‐1, ERK and p‐ERK were decreased. In HG+IL‐1β‐treated rat synovial fibroblasts, PSTPIP2 protein expression was decreased compared to normal glucose (NG)‐treated cells. Levels of TNF‐α, IL‐6, and IL‐1β, as well as expression of MMP‐3, MMP‐13, ADAMTS‐5, ICAM‐1, ERK and p‐ERK were increased. After cells were infected with PSTPIP2‐overexpressed lentivirus, levels of TNF‐α, IL‐6, and IL‐1β, and expression of MMP‐3, MMP‐13, ADAMTS‐5, ICAM‐1, ERK and p‐ERK were obviously decreased compared to cells infected with NC lentivirus. In addition, ERK inhibitor U0126 treatment also decreased the TNF‐α, IL‐6, and IL‐1βlevels and MMP‐3, MMP‐13, ADAMTS‐5, ICAM‐1, ERK and p‐ERK expression in HG + IL‐1β treated rat synovial fibroblasts.

**Conclusion:**

Overexpression of PSTPIP2 alleviates synovial inflammation and cartilage injury during DM‐OA progression *via* inhibiting ERK phosphorylation.

## Graphical abstract

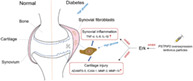

Diabetes could induce synovial inflammation and cartilage injury by stimulating fibroblast‐like synoviocytes with high glucose. Injection of *PSTPIP2* overexpression lentivirus particles could alleviate synovial inflammation and cartilage injury *via* inhibiting ERK phosphorylation.

## Introduction

Diabetes mellitus (DM) is a group of metabolic disorders characterized by hyperglycemia due to insufficient insulin secretion, insulin resistance, or both. As one of the most prevalent diseases, DM occurs in about 8%–9% of the world's population. By 2015, over 415 million adults have been diagnosed with DM, while the number is estimated to increase to 642 million by 2040^1^. The most common forms of DM are type 1 diabetes mellitus (T1DM) and type 2 diabetes mellitus (T2DM). T1DM accounts for about 5%–10% of all DM cases, with a strong genetic predisposition^2^. T2DM accounts for over 90% of all DM cases and leads to multiple complications that cause profound psychological and physical distress to patients^3^. The complications associated with T2DM mainly include the cardiovascular diseases, the renal diseases, the risks of disorders in hepatic, musculoskeletal, and digestive systems, and the incidence of certain cancers^4^. It has been reported that about 36%–75% of diabetic patients suffer from musculoskeletal complications, which sometimes lead to severe disabilities^5^.

Osteoarthritis (OA) is a degenerative joint disease characterized by cartilage loss, subchondral bone remodeling, osteophyte formation and synovial inflammation^6^, ^7^. OA affects approximately 10%–12% of adults and is one of the main causes of disability, bringing a commensurate tremendous burden to individuals and society^8^. Epidemiological investigations have shown that T2DM and OA frequently co‐exist in aged people due to the high prevalence and shared risk factors^9^. Moreover, convinced evidence has demonstrated that patients with T2DM have a higher risk of developing OA^10^, ^11^, ^12^. Several reported mechanisms support that T2DM affects OA progression through hyperglycemia and insulin resistance, which subsequently causing synovitis and cartilage damage in joint tissues^13^. Nevertheless, the detailed molecular mechanism remains largely unclear.

Proline‐serine–threonine phosphatase‐interacting protein 2 (PSTPIP2), also known as macrophage actin‐associated tyrosine phosphorylated protein (MAYP), belongs to the Pombe Cdc15 homology (PCH) family located on chromosome 18^14^. The members of proteins in PCH family have been identified as critical regulators in the cytokinesis, endocytosis, cell adhesion, and motility of macrophages^15^. It has been reported that mutation in the mouse PSTPIP2 gene causes a spontaneous inflammatory response characterized by macrophage infiltration, leading to osteolysis and necrosis in paws and ears^16^. PSTPIP2 deficiency in mice may lead to an expansion of macrophage progenitors, and the enhancement of the responsiveness of mature macrophages to activation stimuli, which together promotes an excessive and sustained response to the autoinflammatory diseases^17^. Moreover, PSTPIP2 deficiency in mice causes osteopenia and bone lesions, and increases the differentiation of multipotent myeloid precursors into osteoclasts^18^. Some recent studies have reported that overexpression of PSTPIP2 could inhibit the proliferation and inflammatory response of fibroblast‐like synoviocytes^19^. In addition, overexpression of PSTPIP2 in rats could attenuate the adjuvant‐induced arthritis (AIA)‐associated bone loss and inflammatory infiltration^20^. However, whether PSTPIP2 takes part in the progression of DM‐OA is still unclear.

Extracellular signal‐regulated kinase (ERK) is a vital protease that can be activated by inflammatory cytokines. The activated ERK regulates a numerous of cellular signaling transduction and is involved in many physiological and disease processes, including OA^21^. Notably, Liu *et al*. found that PSTPIP2 repressed megakaryocyte differentiation by inhibiting ERK signaling^22^. However, what role ERK plays in DM‐OA progression is unknown. The main objectives of this study are to investigate the biological functions of PSTPIP2 in the pathogenesis of DM‐OA, and to explore whether PSTPIP2 affects DM‐OA progression by regulating ERK signaling.

## Materials and Methods

### Construction of Lentivirus Vectors Overexpressing PSTPIP2

To construct PSTPIP2‐overexpressing lentivirus vectors, PSTPIP2‐overexpressing plasmids were constructed by cloning rat PSTPIP2 cDNA into the PLJM1 lentivirus vector (Addgene, Cambridge, CA, USA). The PLJM1‐ PSTPIP2 plasmids or its empty vector plasmids were transfected into HEK293T cells using Lipofectamine 2000 (Invitrogen, Carlsbad, CA, USA) to produce lentivirus particles, according to the manufacturer's protocols.

### Establishment of DM‐OA Rat Model and Treatment

All animal experimental procedures were approved by the Ethical Committee of the 2nd Affiliated Hospital of Harbin Medical University and in accordance with the Guide for the Care and Use of Laboratory Animals (NIH Publication Vol 25, No. 28 revised 1996).

To establish DM‐OA rat model *in vivo*, eight‐week‐old Sprague Dawley male rats were purchased from Liaoning Changsheng Biotechnology Co., Ltd. (Benxi, China). Rats were housed in standard cages under general conditions (temperature of 22 ± 1 °C; humidity of 45%–55%; 12‐h light and 12‐h dark) with free access to food and water. Rats were randomly divided into the following four groups: control (Ctrl) group; DM‐OA group; DM‐OA + lentivirus (LV)‐negative control (NC) group; DM‐OA + LV‐PSTPIP2 group. At first, rats in the experimental groups received an intraperitoneal injection of streptozotocin (STZ, 30 mg/kg, dissolved in citrate buffer) to establish DM model, while rats in the Ctrl group received citrate buffer only. Three days after STZ injection, fasting blood glucose levels in rats of the experimental groups were measured. Rats with blood glucose levels >16.7 mmol/L were defined as diabetic rats and chosen for further experiments. All diabetic rats were fed with a high‐fat diet and received an intra‐articular injection of monoiodoacetate (MIA, 3 mg/rat, dissolved in saline), while control rats received saline only. The next day, rats in the DM‐OA + LV‐PSTPIP2 group received an intra‐articular injection of LV‐PSTPIP2 (1 × 10^8^ TU/mL), while rats in the DM‐OA + LV‐NC group received an equal amount of lentivirus‐vector. Lentivirus particles were supplemented into the rats once a week. Four weeks after MIA injection, rats were euthanized. Serum, knee joints and synovial tissues were removed and stored in −80°C for further testing.

### Cell Culture, Infection and Treatment

To establish DM‐OA cell model *in vitro*, rat synovial fibroblasts were purchased from iCell Bioscience Inc. (Shanghai, China) and maintained in DMEM medium (Gibco Life Technologies, Carlsbad, CA, USA) supplemented with 1% fibroblast growth supplement (Sciencell, Carlsbad, CA, USA) and 10% fetal bovine serum (Sigma‐Aldrich, St Louis, MO, USA). Cells were infected with lentivirus particles to overexpress PSTPIP2. Two days after lentivirus infection, cells were added with normal glucose (5.5 mmol/L, Aladdin regents, Shanghai, China), high glucose (25 mmol/L), IL‐1β (5 ng/mL, MedChemExpress, Monmouth Junction, NJ, USA) or ERK inhibitor U0126 (10 μM, MedChemExpress) according to the group information. Two days later, cells were collected for further testing.

### Real‐Time Polymerase Chain Reaction (RT‐PCR)

To detect PSTPIP2 messenger RNA (mRNA) level in joint tissues of rats, total RNA was extracted using TRIpure reagent (BioTeke Corporation, Beijing, China). Complementary DNA (cDNA) was synthesized using Reverse Transcription M‐MLV (BioTeke). Real‐time PCR was performed in Exicycler^96^ (Bioneer Corporation, Daejeon, Korea) with Power SYBR Green PCR Master Mix (BioTeke) according to the supplier's instructions. Primers used for PSTPIP2 amplification were: forward 5’‐GCAGCATTGAGAAGGACA‐3′ and reverse 5′‐CGCTTAGGAACTGGGAGA‐3′. Primers used for β‐actin amplification were: forward 5′‐GGAGATTACTGCCCTGGCTCCTAGC‐3′ and reverse 5′‐GGCCGGACTCATCGTACTCCTGCTT‐3′. β‐actin was used as an internal control. The relative mRNA levels were analyzed relative to threshold cycle values (ΔCt), then calculated using the 2^‐ΔΔCt^ method.

### Western Blotting (WB)

To detect PSTPIP2, matrix metalloproteinase (MMP)‐3, MMP‐13, aggrecanase‐2 (ADAMTS‐5), intercellular cell adhesion molecule (ICAM)‐1, ERK and phospho‐ERK (p‐ERK) protein expression in rat synovial tissues or synovial fibroblasts, total protein was extracted using cell lysis buffer for Western and IP (Beyotime, Shanghai, China) and then quantified using BCA protein assay kit (Beyotime). Equal amount of protein of each sample was separated by 8% or 11% sodium dodecyl sulphate‐polyacrylamide gel electrophoresis (SDS‐PAGE) and transferred into polyvinylidene difluoride (PVDF) membranes (Millipore, Billerica, MA, USA). Membranes were blocked with 5% skim milk and then incubated with primary antibodies including PSTPIP1 rabbit polyclonal antibody (ABclonal Biotechnology, Wuhan, China; dilution at 1:1000), ADAMTS5 rabbit polyclonal antibody (ABclonal; dilution at 1:1000), ICAM‐1 rabbit polyclonal antibody (ABclonal; dilution at 1:1000), MMP‐3 rabbit monoclonal antibody (ABclonal; dilution at 1:1000), MMP‐13 rabbit polyclonal antibody (ABclonal; dilution at 1:1000), ERK1/2 rabbit polyclonal antibody (Affinity, San Diego, California, USA; dilution at 1:1000), phospho‐ERK1/2 (Thr202/Tyr204) rabbit polyclonal antibody (Affinity; dilution at 1:1000) and β‐actin (Santa Cruz Biotechnology, CA, USA; dilution at 1:1000) overnight at 4°C. After washing with PBS‐tween for three times, membranes were subsequently incubated for 1 h at 37°C with goat anti‐mouse IgG (Beyotime; dilution at 1:3000) or goat anti‐rabbit IgG (Beyotime; dilution at 1:3000). ECL solution (Beyotime) was added for detection and Gel‐Pro‐Analyzer system was used to analyze the grayscale value of protein bands.

### Hematoxylin/Eosin (HE) Staining

To observe the histological changes in the synovium, rat knee joints were dissected and then decalcified using JYBL‐I solution (Solarbio Science & Technology, Beijing, China). The decalcified samples were dehydrated in ascending grades of alcohol and embedded in paraffin. Specimens were then sectioned into 5 μm thickness slices and stained with hematoxylin (Solarbio) and eosin (Sangon Biotech, Shanghai, China) according to the supplier's protocols. Microscopic images were collected using a light microscopy (DP73; Olympus Corporation, Tokyo, Japan).

### Synovitis Score

The histopathological synovitis score was applied to assess the synovial inflammation. Synovitis score was evaluated according to the status of tissue architecture and pathological change to be observed in HE staining results^23^. The scores of three features (enlargement of lining cell layer, cellular density of synovial stroma, leukocytic infiltrate) were assessed (from 0, absent to 3, strong) and total score was summed up. Higher synovitis score indicates more severe synovial inflammation happened in joints.

### Safranin O/Fast Green Staining

To observe the histological changes in the cartilage, rat knee joint sections were stained with safranin O and fast green according to the supplier's protocols. Microscopic images were collected using a light microscopy.

### Osteoarthritis Research Society International (OARSI) Score

OARSI score was formulated by OARSI in 1998 to standardize the assessment of OA histopathology, according to Safranin O/fast green staining results. OARSI score represents a combined assessment based on the severity and extent of OA in the articular cartilage while the total score ranges from 0 to 6. The specific standards were consistent with the previous report^24^. Higher OARSI score indicates more severe cartilage injury happened in joints.

### Enzyme Linked Immunosorbent Assay (ELISA)

To detect the levels of proinflammatory cytokines tumor necrosis factor (TNF)‐α, interleukin (IL)‐6, and IL‐1β in synovial tissues or synovial fibroblasts, ELISA was performed. TNF‐α, IL‐6 and IL‐1β levels were measured using commercially available kits (USCN Life Science, Wuhan, China) according to the manufacturer's instructions.

### Expression of Related Proteins Involved in Synovial Inflammation, Cartilage Injury and ERK Signaling

To detect the expression of related proteins involved in synovial inflammation, cartilage injury and ERK signaling, the following parameters were chosen for evaluation.

### TNF‐α

TNF‐α is a major inflammatory cytokine that involved in maintenance and homeostasis of the immune system, inflammation, and host defense. In this experiment, the expression of TNF‐α was detected by ELISA.

### IL‐6

IL‐6 is a prototypical cytokine with functional pleiotropy and plays an important role in inflammation and host defense. In this experiment, the expression of IL‐6 was detected by ELISA.

### IL‐1β

The proinflammatory cytokine IL‐1β plays critical role in inflammatory and autoimmune diseases. In this experiment, the expression of IL‐1β was detected by ELISA.

### MMP‐3

MMP‐3 is a cartilage‐degrading enzyme that associates with the degradation of type II collagen and aggrecan. It is an important catabolic factor that reflects cartilage damage. In this experiment, the expression of MMP‐3 was detected by WB.

### MMP‐13

MMP‐13 is a product of the chondrocytes that reside in the cartilage and is involved in degrading type II collagen in articular cartilage. It is a key enzyme responsible for the degenerative changes in cartilage. In this experiment, the expression of MMP‐13 was detected by WB.

### ADAMTS‐5

ADAMTS‐5 is an enzyme responsible for cleaving aggrecan, the major proteoglycan in articular cartilage. It is an indicator of cartilage matrix destruction. In this experiment, the expression of ADAMTS‐5 was detected by WB.

### ICAM‐1

ICAM‐1 is an inducible cell surface glycoprotein that associates with leukocyte migration, adhesion and function. It promotes monocyte recruitment into the synovial tissue and participates cartilage damage. In this experiment, the expression of ADAMTS‐5 was detected by WB.

### ERK and p‐ERK

ERK is activated by phosphorylation in activation loop to induce specific cell responses. ERK phosphorylation positively links with the pathogenesis of OA. In this experiment, the expression of ERK and p‐ERK was detected by WB.

### Statistical Analysis

All data were shown as mean ± standard derivation (SD). Statistical analysis was performed using GraphPad Prizm V8.0.2 (GraphPad Software, CA, USA). Normally distributed data were analyzed by one‐way ANOVA with Tukey's post‐hoc test. Nonparametric data were analyzed by Kruskal–Wallis test. Differences were considered significant at *P* < 0.05.

## Results

### Expression of PSTPIP2 in DM‐OA Rats after Treatment with LV‐PSTPIP2 or LV‐NC

**Fig. 1:**
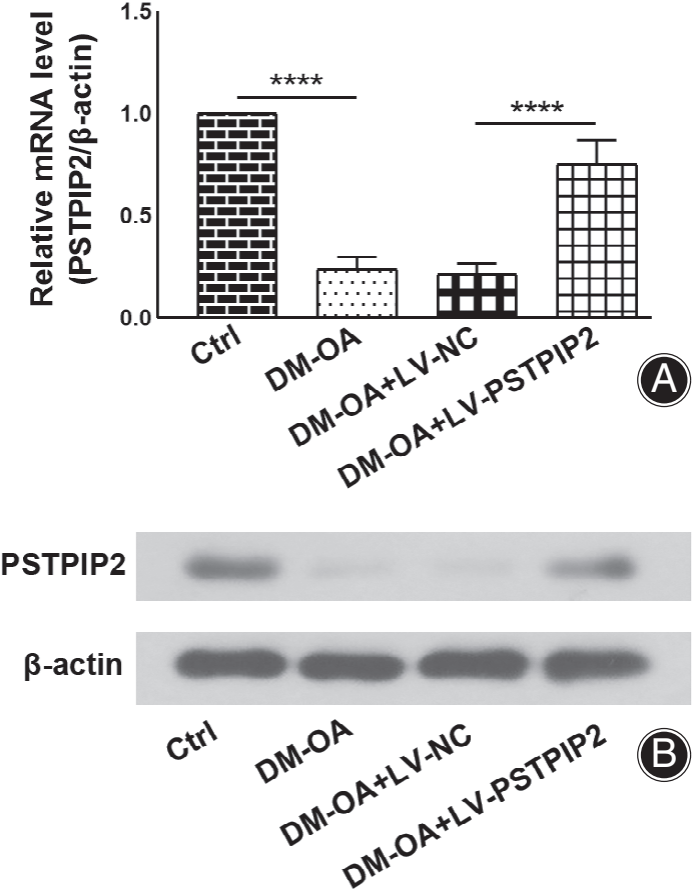
Detection of PSTPIP2 expression *in vivo*. Eight‐week old male SD rats were induced with DM‐OA by streptozotocin injection/high fat diet and intra‐articular injection of monoiodoacetate. Overexpression of PSTPIP2 *in vivo* was constructed by intra‐articular injection of lentivirus vectors. (A) Quantitative real‐time PCR analysis of PSTPIP2 mRNA levels in synovial tissues for all groups. (B) Western blotting analysis of PSTPIP2 protein in synovial tissues for all groups. ****P < 0.0001. *n* = 6. Ctrl, control; DM‐OA, diabetes mellitus‐osteoarthritis; LV‐NC, negative control lentivirus transfected; LV‐PSTPIP2, PSTPIP2 overexpression lentivirus transfected.

In order to investigate the role of PSTPIP2 in DM‐OA progression, we established a rat DM‐OA model. PSTPIP2 expression at the mRNA level and protein level was detected by RT‐PCR and WB respectively. As shown in Fig. 1A, the relative PSTPIP2 level in DM‐OA rats was significantly decreased compared to the control rats (0.23 ± 0.06 *vs* 1.00 ± 0.00, *P* < 0.0001). After treatment with LV‐PSTPIP2 or LV‐NC, the relative PSTPIP2 mRNA level was significantly increased in the DM‐OA + LV‐PSTPIP2 group compared to the DM‐OA + LV‐NC group (0.75 ± 0.12 *vs* 0.21 ± 0.05, *P* < 0.0001).

Similarly, PSTPIP2 expression was found obviously reduced in the DM‐OA group compared to the control group. Reversely, PSTPIP2 expression was markedly increased in the DM‐OA + LV‐PSTPIP2 group compared to the DM‐OA + LV‐NC group (Fig. 1B). These results indicated that PSTPIP2 expression was down‐regulated in DM‐OA rats and re‐increased after treatment with LV‐PSTPIP2.

### Overexpression of PSTPIP2 Inhibited Synovial Inflammation in DM‐OA Rats

**Fig. 2:**
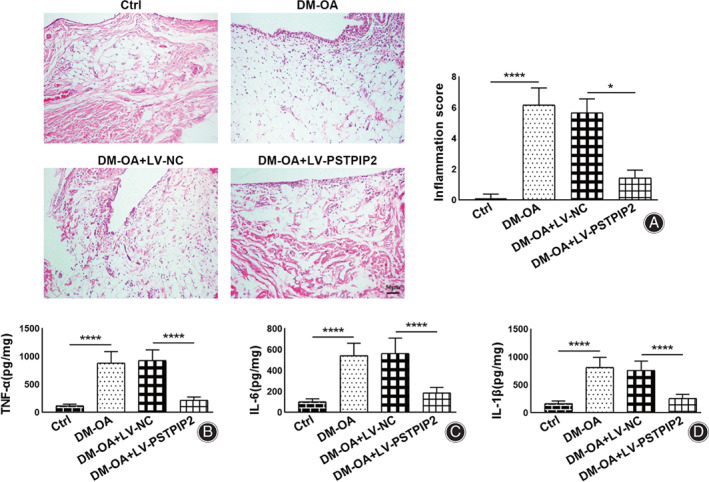
PSTPIP2 overexpression inhibits synovial inflammation in DM‐OA rats. (A) Representative hematoxylin/eosin‐stained synovial histological images and synovial inflammation scores for all groups. (B–D) ELISA analysis of TNF‐α (B), IL‐6 (C) and IL‐1β (D) levels in synovial tissues for all groups. *P < 0.05, ****P < 0.0001. *n* = 6. Ctrl, control; DM‐OA, diabetes mellitus‐osteoarthritis; LV‐NC, negative control lentivirus transfected; LV‐PSTPIP2, PSTPIP2 overexpression lentivirus transfected.

Synovial inflammation was evaluated according to two aspects. One is the assessment of synovitis score, the other is the detection on the expressions of synovial pro‐inflammatory cytokines, including TNF‐α, IL‐6 and IL‐1β. As shown in Fig. 2A, the hematoxylin/eosin staining results showed that in the control group, the cartilage was smooth and complete, with regularly arranged chondrocytes. No hyperplasia or fibrosis was observed in the synovium. In the DM‐OA group, cartilage structure injury, synovial hyperplasia and inflammatory cell infiltration were obviously observed. Similar results were observed in the DM‐OA + LV‐NC group. In the DM‐OA + LV‐PSTPIP2 group, the degree of cartilage structure injury, synovial hyperplasia and inflammatory cell infiltration were obviously relieved. Consistently, the synovitis score in the DM‐OA group was significantly increased compared to the control group (6.17 ± 1.11 *vs* 0.08 ± 0.29, *P* < 0.0001). In contrast, synovitis score in the DM‐OA + LV‐PSTPIP2 group was significantly decreased compared to the DM‐OA + LV‐NC group (1.42 ± 0.51 *vs* 5.67 ± 0.89, *P* < 0.05).

Moreover, we detected the levels of proinflammatory cytokines TNF‐α, IL‐6 and IL‐1β using ELISA to reflect inflammatory response. The results showed that the TNF‐α (880.74 ± 206.23 *vs* 116.86 ± 28.82 pg./mg, *P* < 0.0001), IL‐6 (541.01 ± 118.38 *vs* 101.01 ± 28.25 pg./mg, *P* < 0.0001), and IL‐1β (812.66 ± 178.75 *vs* 164.53 ± 49.00 pg./mg, *P* < 0.0001) levels were significantly raised in the DM‐OA group compared to the Ctrl group. Reversely, the levels of TNF‐α (216.19 ± 58.77 *vs* 926.02 ± 191.73 pg./mg, *P* < 0.0001), IL‐6 (185.95 ± 52.49 *vs* 559.83 ± 147.71 pg./mg, *P* < 0.0001), and IL‐1β (256.06 ± 73.85 *vs* 760.07 ± 164.79 pg./mg, *P* < 0.0001) levels were dramatically decreased in the DM‐OA + LV‐PSTPIP2 group compared to the DM‐OA + LV‐NC group (Fig. 2B–D). These results indicated that overexpression of PSTPIP2 effectively inhibited synovial inflammation in DM‐OA rats.

### Overexpression of PSTPIP2 Attenuated Cartilage Injury in DM‐OA Rats

**Fig. 3:**
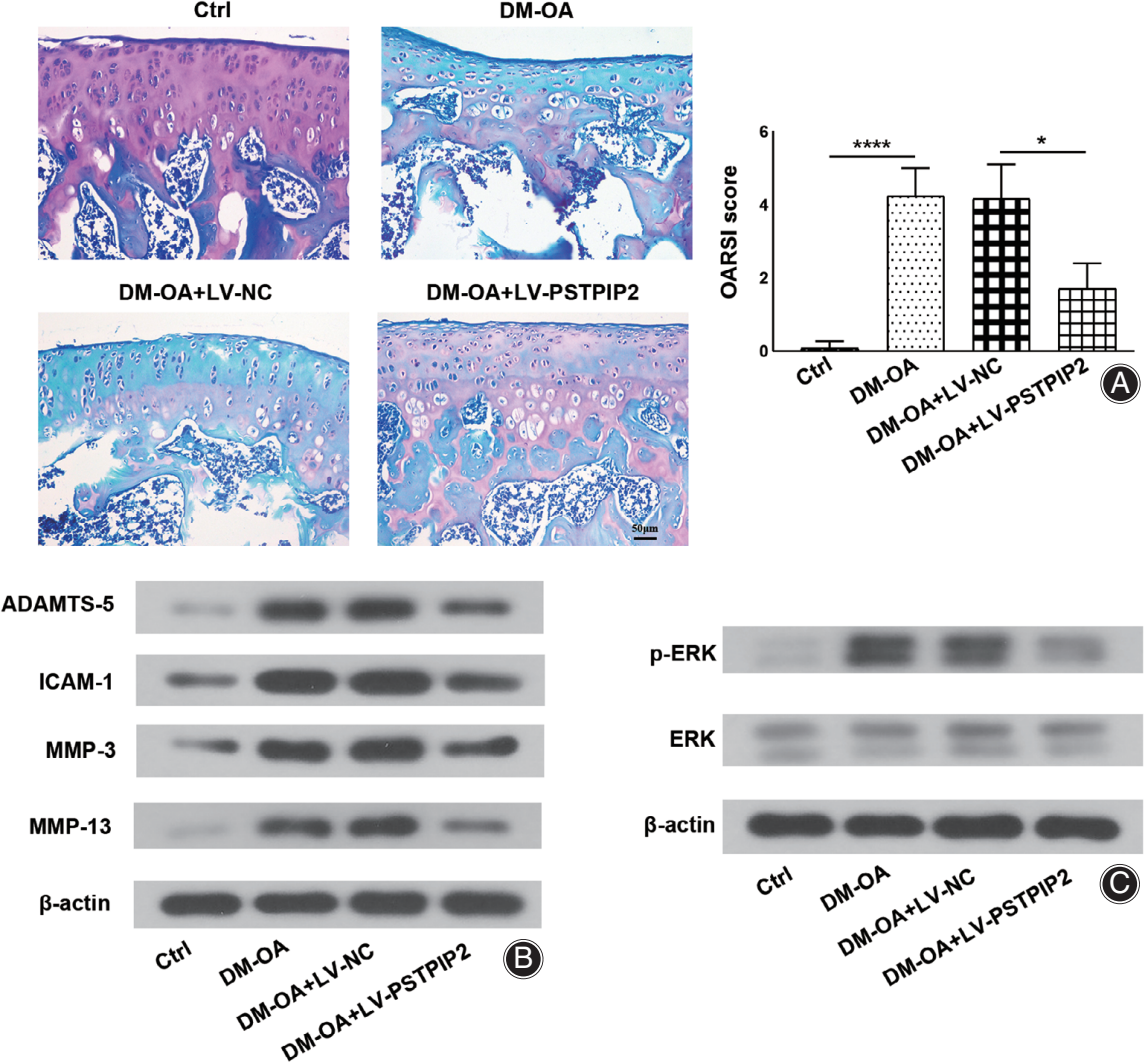
PSTPIP2 overexpression attenuates cartilage injury and inhibits ERK phosphorylation in DM‐OA rats. (A) Representative safranin O/fast green‐stained cartilage histological images and OARSI scores for all groups. (B) Western blotting analysis of MMP‐3, MMP‐13, ADAMTS‐5, ICAM‐1 and β‐actin proteins in synovial tissues for all groups. (C) Western blotting analysis of p‐ERK, ERK and β‐actin proteins in synovial tissues for all groups. *P < 0.05, ****P < 0.0001. *n* = 6. Ctrl, control; DM‐OA, diabetes mellitus‐osteoarthritis; LV‐NC, negative control lentivirus transfected; LV‐PSTPIP2, PSTPIP2 overexpression lentivirus transfected.

Cartilage injury was evaluated according to two aspects. One is the assessment of OARSI score, the other is the detection on the expressions of matrix metalloproteinases MMP‐3 and MMP‐13, aggrecanase ADAMTS‐5 and adhesion molecule ICAM‐1. Safranin‐O/fast‐green staining was performed to observe cartilage damage (basophile chondrocytes were stained red by safranin‐O, and eosinophilic bone cells were stained blue by fast‐green). As shown in Fig. 3A, in the control group, the chondrocytes were widely distributed and the cartilage was intact and smooth without injuries. In the DM‐OA and DM‐OA + LV‐NC groups, few chondrocytes were observed and the articular cartilage was obviously injured. In the DM‐OA + LV‐PSTPIP2 group, the chondrocytes were increased again and the cartilage injury was evidently attenuated compared to the DM‐OA + LV‐NC group. Consistently, OARSI score in the DM‐OA group was markedly increased compared to the control group (4.25 ± 0.75 *vs* 0.17 ± 0.26, *P* < 0.0001), whereas OARSI score in the DM‐OA + LV‐PSTPIP2 group was significantly decreased compared to the DM‐OA + LV‐NC group (1.71 ± 0.69 *vs* 4.17 ± 0.94, *P* < 0.05).

Moreover, we detected the expression of MMP‐3, MMP‐13, ADAMTS‐5, and ICAM‐1 using WB to assess cartilage degradation. The protein levels of MMP‐3, MMP‐13, ADAMTS‐5 and ICAM‐1 were obviously increased in DM‐OA rats compared to control rats. Simultaneously, the protein levels of these factors were markedly decreased in DM‐OA + LV‐PSTPIP2 group compared to DM‐OA + LV‐NC group (Fig. 3B). The data suggested that overexpression of PSTPIP2 attenuated cartilage injury in DM‐OA rats.

### Overexpression of PSTPIP2 Inhibited ERK Phosphorylation in DM‐OA Rats

To clarify the mechanism of PSTPIP2‐mediated protective effect on DM‐OA in rats, we detected ERK and p‐ERK to evaluate the effect or PSTPIP2 on ERK activation. The results of WB analysis showed that in the DM‐OA group, ERK phosphorylation level was dramatically increased compared to the Ctrl group. In addition, ERK phosphorylation level was decreased in the DM‐OA + LV‐PSTPIP2 group compared to DM‐OA + LV‐NC group (Fig. 3C), indicating that PSTPIP2 may exert its effects by inhibiting ERK activation.

### Overexpression of PSTPIP2 Inhibited ERK Phosphorylation in HG/IL‐1β‐Treated Rat Synovial Fibroblasts

**Fig. 4:**
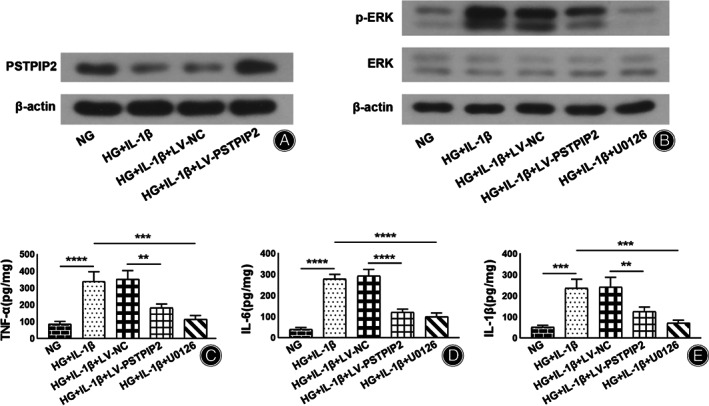
PSTPIP2 overexpression inhibits ERK phosphorylation and restores inflammatory cytokine levels in HG/IL‐1β‐treated rat synovial fibroblasts. (A) Rat synovial fibroblasts were infected by lentivirus vectors and treated by NG (5.5 mM) or HG (25 mM) and IL‐1β (5 ng/mL). Western blotting analysis of PSTPIP2 protein in synovial fibroblasts for all groups. (B) ERK inhibitor U0126 was added to the treated synovial fibroblasts. Western blotting analysis of p‐ERK, ERK and β‐actin proteins in synovial fibroblasts for all groups. (C–E) ELISA analysis of TNF‐α (C), IL‐6 (D) and IL‐1β (E) levels in synovial fibroblasts for all groups. **P < 0.01, ***P < 0.001, ****P < 0.0001. *n* = 3. NG, normal glucose; HG, high glucose; LV‐NC, negative control lentivirus transfected; LV‐PSTPIP2, PSTPIP2 overexpression lentivirus transfected.

To further investigate the role of PSTPIP2 *in vitro*, a cell model was established. The results of WB showed that in HG/IL‐1β‐treated rat synovial fibroblasts, the expression of PSTPIP2 was down‐regulated compared to NG‐treated cells. After infected with PSTPIP2‐overexpressed lentivirus, PSTPIP2 expression was obviously up‐regulated compared with its vector control (Fig. 4A). Moreover, to better interpret the effect of PSTPIP2 on ERK, ERK inhibitor U0126 was added to the treated cells as a positive control. The results showed that U0126 inhibited the elevated ERK phosphorylation level in HG/IL‐1β‐treated rat synovial fibroblasts (Fig. 4B). These results implicated that PSTPIP2 inhibited ERK phosphorylation in HG/IL‐1β‐treated rat synovial fibroblasts.

### Both Overexpression of PSTPIP2 and Inhibition of ERK Reduced Inflammatory Cytokine Levels in HG/IL‐1β‐Treated rat Synovial Fibroblasts

The levels of proinflammatory cytokines TNF‐α, IL‐6 and IL‐1β in HG/IL‐1β‐treated rat synovial fibroblasts were measured by ELISA to reflect inflammatory response *in vitro*. The results showed that In HG + IL‐1β‐treated rat synovial fibroblasts, the levels of TNF‐α (339.89 ± 56.43 *vs* 85.13 ± 16.79 pg/mg, *P* < 0.0001), IL‐6 (277.50 ± 21.85 *vs* 39.91 ± 8.08 pg/mg, *P* < 0.0001), and IL‐1β (235.55 ± 43.29 *vs* 52.25 ± 7.98 pg/mg, *P* < 0.001) were significantly increased. After cells were infected with LV‐PSTPIP2, the levels of TNF‐α (181.09 ± 23.84 *vs* 352.19 ± 50.66 pg/mg, *P* < 0.01), IL‐6 (119.46 ± 16.08 *vs* 291.88 ± 31.77 pg/mg, *P* < 0.0001), and IL‐1β (126.06 ± 20.62 *vs* 241.09 ± 46.37 pg/mg, *P* < 0.01) were significantly decreased compared to cells infected with LV‐NC. Also, after treatment with U0126, the levels of TNF‐α (115.11 ± 21.82 *vs* 339.89 ± 56.43 pg/mg, *P* < 0.001), IL‐6 (99.89 ± 17.30 *vs* 277.50 ± 21.85 pg/mg, *P* < 0.0001), and IL‐1β (71.40 ± 13.22 *vs* 235.55 ± 43.29 pg/mg, *P* < 0.001) were significantly decreased as well. These results showed that both overexpression of PSTPIP2 and inhibition of ERK inhibited inflammatory response in HG/IL‐1β‐treated rat synovial fibroblasts (Fig. 4C–E).

### Both Overexpression of PSTPIP2 and Inhibition of ERK Restored Cartilage Synthesis‐Related Factor Levels in HG/IL‐1β‐Treated Rat Synovial Fibroblasts

**Fig. 5:**
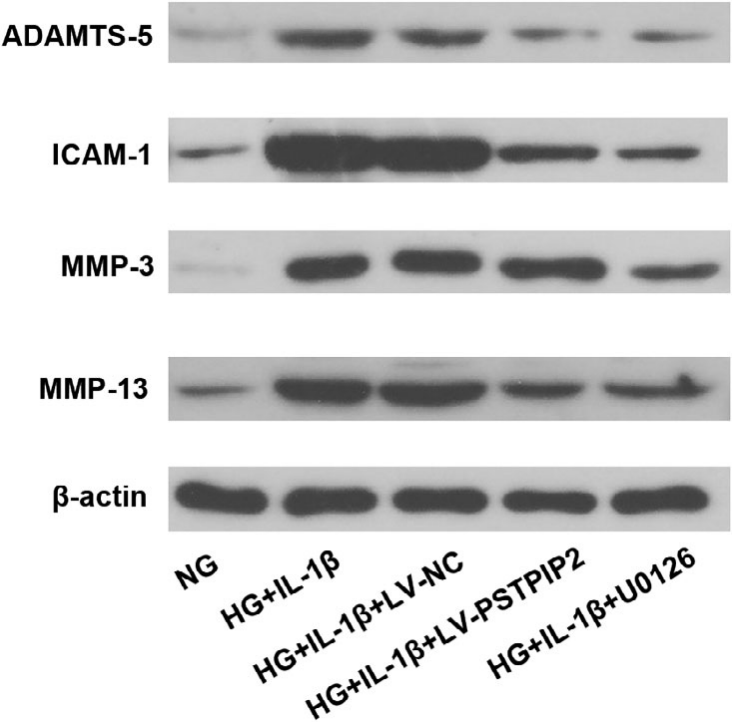
PSTPIP2 overexpression restores cartilage synthesis‐related factor levels in HG/IL‐1β‐treated rat synovial fibroblasts. Western blotting analysis of MMP‐3, MMP‐13, ADAMTS‐5, ICAM‐1 and β‐actin proteins in synovial fibroblasts for all groups. *n* = 3. NG, normal glucose; HG, high glucose; LV‐NC, negative control lentivirus transfected; LV‐PSTPIP2, PSTPIP2 overexpression lentivirus transfected.

The expression of cartilage degradation‐related factors, ADAMTS‐5, ICAM‐1, MMP‐3 and MMP‐13 was detected by WB to reflect cartilage injury *in vitro*. The results showed that In HG + IL‐1β‐treated rat synovial fibroblasts, the expression of ADAMTS‐5, ICAM‐1, MMP‐3 and MMP‐13 was obviously up‐regulated compared to NG‐treated cells. On the contrary, the expression of ADAMTS‐5, ICAM‐1, MMP‐3 and MMP‐13 was down‐regulated in the HG + IL‐1β + LV‐PSTPIP2 group compared to the HG + IL‐1β + NC group. Also, the expression of these factors was down‐regulated in the HG + IL‐1β + U0126 group compared to the HG + IL‐1β group. These data implied that both overexpression of PSTPIP2 and inhibition of ERK reduced cartilage degradation‐related factors in HG/IL‐1β‐treated rat synovial fibroblasts (Fig. 5).

## Discussion

Recently, the systemic role of metabolic syndrome in OA pathophysiology has been partly understood^25^, ^26^, ^27^, but still more studies need to be invested to better interpret the metabolic syndrome‐associated OA phenotype. It has been reported that PSTPIP2 could suppress the differentiation of osteoclasts^22^ and inhibit the inflammatory response of fibroblast‐like synoviocytes^19^. However, whether PSTPIP2 plays a protective role in DM‐OA progression is unknown. In this study, we investigated the effect of PSTPIP2 on DM‐OA *in vivo* and *in vitro*.

We induced T2DM in rats by a high‐fat diet and an intraperitoneal injection of STZ, then induced OA by an intra‐articular injection of MIA. To evaluate the function of PSTPIP2, we constructed PSTPIP2‐overexpression lentivirus and the gave it to the DM‐OA rats by intra‐articular injection. We found that induction of DM‐OA caused profound synovial inflammation and cartilage damage in rats, which were substantially but not completely attenuated by overexpression of PSTPIP2.

Up‐regulation of inflammation in synovium is identified as a root cause of OA secondary to DM, involving the changes of various inflammatory mediators^28^. Inflammatory cytokines involved in innate immune system, including TNFα, IL‐6 and IL‐1β, are found at elevated levels in DM‐associated OA^29^. Therefore, blocking the inflammatory response may be a reasonable basis for the exploitation of attenuating DM‐OA progression. Many studies have been dedicated to this theory. For example, Qu *et al*. suggested that SIRT2 suppressed the inflammatory response and oxidative stress in diabetic OA *via* the deacetylation of H3^30^. In addition, Yang *et al*. illustrated that carnosine attenuates the development of T2DM‐induced OA and suppresses the inflammatory response *via* reactive oxygen species (ROS)/nuclear factor‐kappaB (NF‐κB) pathway^31^. Analogously, our results demonstrated that overexpression of PSTPIP2 reduced the release of inflammatory cytokines TNFα, IL‐6 and IL‐1β in the synovium of experimental rats, indicating that PSTPIP2 inhibited synovial inflammation in the DM‐OA progression.

Production of matrix metalloproteinases (MMPs) is a key characteristic feature of OA cartilage degeneration, which may compromise the integrity of the extracellular cartilaginous matrix of articular cartilage in OA pathogenesis^32^. Among the MMPs, MMP‐3 and MMP‐13 are the two representative proteases in regulating DM‐OA progression^31^. In this study, we found that MMP‐3 and MMP‐13 were significantly up‐regulated in the synovial tissues of DM‐OA rats. Overexpression of PSTPIP2 partially restored the protein expressions of MMP‐3 and MMP‐13.

MMPs and aggrecanases are thought to play key roles in OA through degradation of extracellular matrix type II collagen and aggrecan^33^, ^34^. Aggrecanases belong to the adamalysin with thrombospondin type 1 motif (ADAMTS) family, of which the members are characterized by disintegrins and metalloproteases with thrombospondin motifs^35^. One of the most efficient aggrecanases related to OA disease progression is ADAMTS‐5^36^. In this study, we also found that ADAMTS‐5 was significantly up‐regulated in DM‐OA rats. Analogously, overexpression of PSTPIP2 partially inhibited the expression of ADAMTS‐5.

Intercellular adhesion molecule‐1 (ICAM‐1), a cell‐surface adhesion molecule secreted by endothelial cells, is reported elevated in patients with distinct variants of rheumatoid synovitis^37^. Gui *et al*. demonstrated that ICAM‐1 is essential for triggering a vicious cycle of inflammation within the joint in a T2DM rat model, which subsequently drives the articular cartilage degradation during early OA progression^38^. Intriguingly, our results also showed that overexpression of PSTPIP2 could reduce the expression of ICAM‐1.

As overexpression of PSTPIP2 effectively alleviated synovial inflammation and reduced cartilage injury in DM‐OA rats, we further explored the underlying mechanism involved. Extracellular signal‐regulated kinase (ERK) has been implicated in the insulin resistance progress associated with obesity and T2DM. Thus, targeting the ERK signaling pathway is regarded as a potential strategy for treating T2DM^39^. Besides, targeting ERK signaling pathway has also been reported to inhibit extracellular matrix calcification and protect articular cartilage in OA. However, no reports have indicated the change of ERK in DM‐OA progression. Notably, in our study, we showed that the phosphorylation level of ERK is obviously increased in DM‐OA rats. Moreover, PSTPIP2 markedly decreased the ERK phosphorylation level.

To further clarify the function of PSTPIP2, we treated rat synovial fibroblasts with HG and IL‐1β to imitate DM‐OA progression *in vitro*. ERK inhibitor U0126 was used as a positive control^40^. Consistently, we found that PSTPIP2 overexpression inhibited ERK phosphorylation *in vitro*. Both PSTPIP2 overexpression and U0126 inhibited the release of inflammatory factors TNFα, IL‐6 and IL‐1β, and decreased the expressions of cartilage synthesis‐related molecules MMP‐3, MMP‐13, ADAMTS‐5 and ICAM‐1 from synovial fibroblasts.

## Limitations

Regrettably, some limitations exist in the current study. These findings lack the support of clinical evidence. The data is preliminary and the mechanism investigation is inadequate. Still more evidence should be invested to support our statement more convincingly.

### Conclusion

**Fig. 6:**
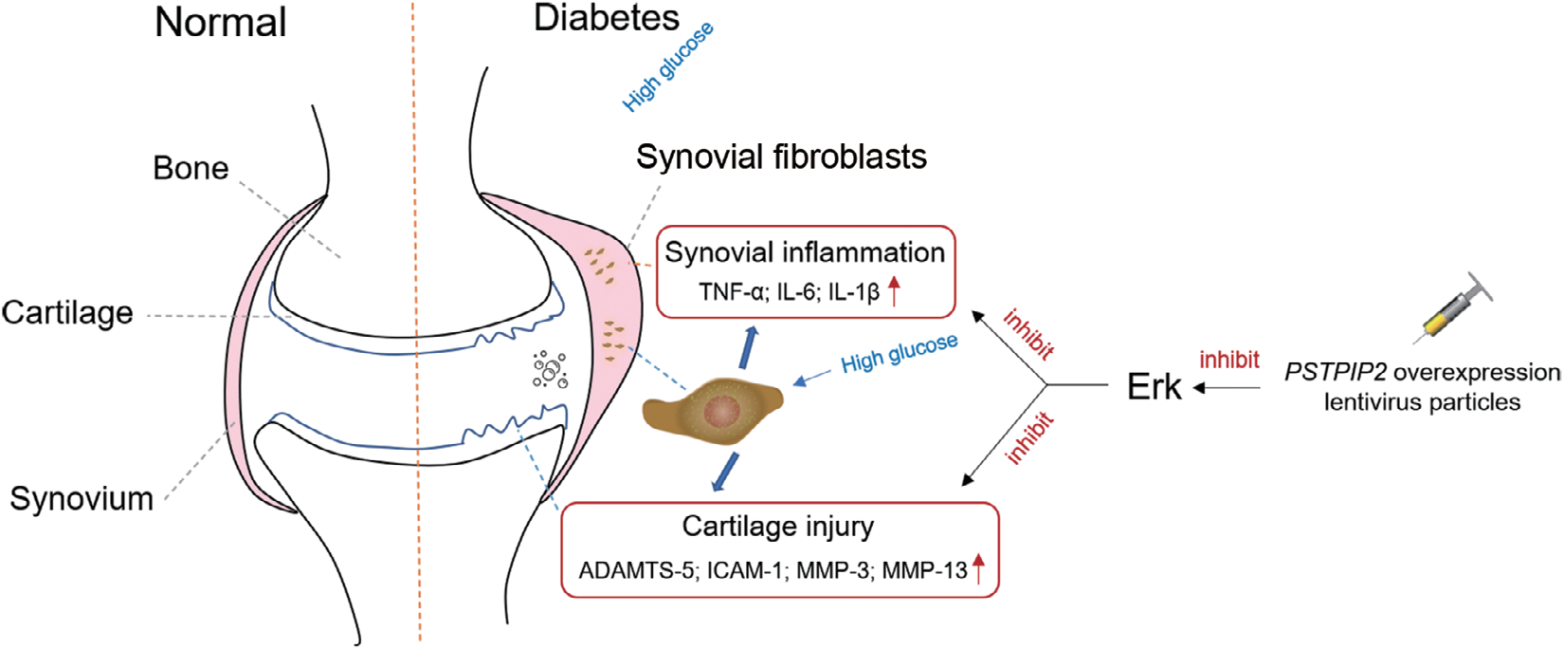
Mechanism diagram of PSTPIP2 overexpression alleviating DM‐OA progression.

In summary, we suggested that PSTPIP2 overexpression plays a protective role against DM‐OA development in the present study. In the *in vivo* and *in vitro* experiments, we demonstrated that overexpression of PSTPIP2 markedly attenuating synovial inflammation and cartilage injury. Moreover, the way that PSTPIP2 exerts its effect against DM‐OA is possibly through inhibition of ERK phosphorylation. The mechanism of regulating network is shown in the illustrative diagram (Fig. 6). These findings indicated that PSTPIP2 may represent a novel therapeutic target in the treatment of DM‐OA progression.

#### Authorship Declaration

All authors listed meet the authorship criteria according to the latest guidelines of the International Committee of Medical Journal Editors. All authors are in agreement with the manuscript.
